# The request for assisted suicide – a challenging topic to teach in medical education. Project report on a compulsory elective course at the Aachen University Hospital

**DOI:** 10.3205/zma001755

**Published:** 2025-06-16

**Authors:** Alexandra Scherg, Miriam Wegmann, Thekla Biersching, Daniel Fink, Martin Lemos, Frank Elsner

**Affiliations:** 1 RWTH Aachen University, Medical Faculty, Department of Palliative Medicine, Aachen, Germany; 2 University of Duisburg-Essen, University Hospital Essen, Department of Palliative Medicine, Essen, Germany; 3 RWTH Aachen University, Faculty of Medicine, Audiovisual Media Center, Aachen, Germany

**Keywords:** assisted suicide, palliative medicine, medical education, professional identity formation

## Abstract

**Aim:**

As part of the Erasmus+ project “ELPIS”, a compulsory elective course in palliative medicine on handling wishes to die and the desire for suicide was put into place and offered for the first time at the Clinic for Palliative Medicine at RWTH Aachen University in the 2023 summer semester, both as a classroom-based session and an online session. The aim of this project was to provide students with background knowledge, skills and the opportunity to stake out a position in the ongoing debate on assisted suicide.

**Method:**

The course was initially offered to 15 students in a classroom-based format and entailed 120 minutes during which an introductory lecture and an interactive conversation with a simulated patient were held. A pseudonymized online evaluation took place. In addition to 4 items to collect demographic data, the focus was specifically on capturing personal attitudes toward and knowledge about assisted suicide (6 items). The survey also described the global outcome (4 items) and measured gain in learning on the levels of knowledge, skills and attitude (8 items).

**Results:**

The evaluation showed that assisted suicide is viewed as a form of medical care, but suggests at the same time that there are knowledge deficits concerning end-of-life alternatives. The students’ self-assessed level of preparedness increased as a result of the intervention, while their fear of being confronted with a wish for assisted suicide decreased. The opportunity to take up a dynamic stance using an athletic playing field as an aid was perceived as helpful. The specific outcome evaluation showed a gain in learning in all of the dimensions.

**Conclusion:**

The participants displayed an open attitude toward assisted suicide as a form of medical care. At the same time, they felt unprepared and fearful of the responsibility that comes with receiving requests for assisted suicide. In order to gain a better understanding of the students’ perspectives and fears, a comparative analysis of the digital course and semi-structured student interviews are currently underway.

## Zusammenfassung

**Zielsetzung:**

In der Klinik für Palliativmedizin der RWTH Aachen University wurde im Rahmen des Erasmus+ Projekts „ELPIS“ ein Wahlpflichtfach „Umgang mit Todes- und Suizidwünschen in der Palliativmedizin“ eingerichtet und im Sommersemester 2023 erstmals sowohl als Präsenzveranstaltung als auch digital angeboten. Ziel des Projektes war, den Studierenden Hintergrundwissen, Handlungskompetenz und eine Option zur dynamischen Positionierung zum Thema Assistierter Suizid anzubieten.

**Methodik:**

Der Kurs wurde zunächst für 15 Studierende in Präsenz angeboten. Er umfasst in einem Zeitraum von 120 min einen Impulsvortrag und ein interaktives Gespräch mit einer Simulationspatientin. Es erfolgte eine pseudonymisierte, elektronische Evaluation. Neben 4 Items zu demografischen Daten lag der Fokus insbesondere auf der Beschreibung der persönlichen Haltung und Wissen zum Assistierten Suizid (6 Items). Zusätzlich wurde die Beschreibung des globalen Outcomes (4 Items) und der Messung des Lernzuwachses auf den Ebenen Wissen, Fertigkeiten und Haltung erfragt (8 Items).

**Ergebnisse:**

Die Evaluation zeigt eine Bewertung der Suizidassistenz als ärztliche Aufgabe, lässt aber gleichzeitig Wissensdefizite zu Alternativen am Lebensende vermuten. Die Selbsteinschätzung des Vorbereitetseins steigerte sich durch die Intervention, die Angst vor der Konfrontation mit Suizidwünschen war rückläufig. Das Angebot zur dynamischen eigenen Positionierung am Modell eines Spielfelds wurde als hilfreich wahrgenommen. Die spezifische Outcome-Evaluation zeigte einen Lernzuwachs in allen Dimensionen.

**Schlussfolgerung:**

Die Teilnehmenden zeigen eine offene Haltung zum Assistierten Suizid als Ärztliche Aufgabe. Zeitgleich fühlen sie sich unzureichend vorbereitet und haben Angst vor der Verantwortung, die die Konfrontation mit Suizidwünschen mit sich bringt. Derzeit erfolgt die vergleichende Auswertung der digitalen Lehrveranstaltung, sowie eine Befragung der Studierenden in semistrukturierten Interviews, um ein besseres Verständnis ihrer Wahrnehmung und Ängste zu erlangen.

## Background

In February 2020 a Federal Constitutional Court ruling lifted the ban on assisted suicide services [1], stating that every individual with the capacity to make informed and deliberate decisions, in self-determination, has the right take one’s own life and to seek assistance from third parties to do so. In 2021, as a consequence, the sentence barring physicians from providing any suicide assistance was deleted from the boilerplate version of the German Medical Association’s professional code of conduct, ending the prohibition on assisting another person to commit suicide for medical doctors [2]. For care providers, this new legal situation results in considerable freedom, but also in great responsibility, not least the need to take an ethical and emotional position on how they personally approach another person’s wish to die or commit suicide. Newly licensed physicians should therefore be prepared early on to adequately assume this freedom and responsibility. In terms of teaching, instructors are faced with the challenge of bringing home the weightiness of this responsibility, which their students will later bear in real medical situations, in an educational setting. Among the teaching staff in palliative medicine at German medical schools, the discussion is currently about the extent to which contending with requests for assisted suicide should even be a part of the undergraduate medical curriculum or should rather be assigned to postgraduate training [3]. This paper describes a new seminar at the RWTH Aachen University Hospital on how to respond to suicide requests and which was brought into being as part of the EU Erasmus+ project “ELPIS” (**EL**earning on **P**alliative Care for **I**nternational **S**tudents), KA220-HED-AF6D681C.

## Project description

Seven medical schools participated in the *ELPIS* project: Rome and Bologna (Italy), Pécs (Hungary), Brasov (Romania), Pamplona (Spain), Hamilton (Canada) and Aachen (Germany).

Based on research of the international literature following realist synthesis, the first work package involved identifying the requisites for successful online teaching [4]. Based on these (educational theory, desired effect of technology, integration in overall curriculum), palliative medicine teaching units were developed at each of the universities for specifically chosen topics or learning objectives.

**Figure 1:**
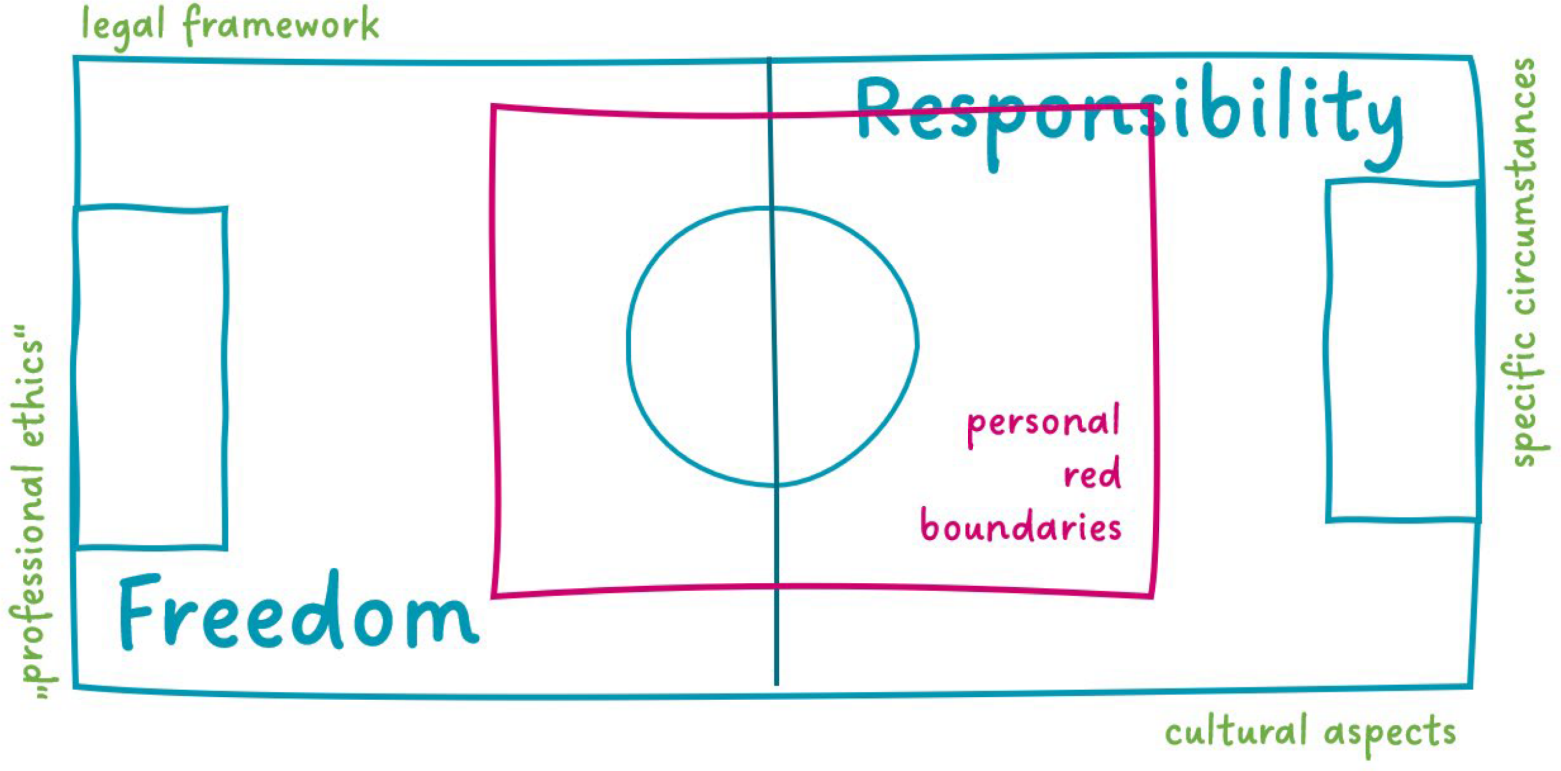
Picture of an athletic playing field to assist in positioning one’s own role

Within the scope of a pilot project, a clinical compulsory elective on responding to death and suicide wishes in palliative medicine was offered for the first time at the Clinic for Palliative Medicine at the RWTH Aachen University Hospital. The course encompassed two 120-minute-long course sessions and an extensive student evaluation. The first session took place in the classroom (in a second session, the content for the same learning objective was presented in an online unit [https://emedia-medizin.rwth-aachen.de/course/view.php?id=573&lang=en]). Both sessions began with an introductory lecture on the legal situation in Germany and a summary of the recommendations given by the German Association for Palliative Medicine (DGP) on how to handle the wish to die and requests for assisted suicide in the hospice and palliative care settings [5]. Furthermore, using an athletic playing field as an aid, the students were offered a way to continually reposition themselves in their approach to assisted suicide (see figure 1 (Fig. 1)). The field can be used, for concrete cases or abstractly, to draw uncrossable red lines, which can naturally shift as new experience is gathered. One example of this could be the one discussed in the course: A patient’s wish for assisted suicide versus a loved one’s feelings. In the simulation that follows in the classroom-based session, students are able to experience and practice confronting a simulated patient’s request for assisted suicide by taking on a double role (doctor and family member). Given the setting of a family celebration, the students slip into the roles of a doctor off-duty in the evening and, at the same time, the patient’s godchild. The godparent, who has advanced lung cancer with brain metastases expresses the wish for assistance with committing suicide. In the online session, a recorded dialogue with the same simulated patient is shown, during which, as the scene unfolds, the attention is turned to the students’ reactions. Over the course of the dialogue, the students are presented with a choice of different options for reacting and responding. The dialogue then takes specific turns depending on their choices, offering at times direct feedback for the students and opening up the opportunity to turn in another direction. At the end of this course there is a pseudonymized evaluation using an online questionnaire (Surveymonkey^®^) with 23 items. In addition to 4 items about demographic data, 6 other items focused in particular on the students’ description of their personal attitudes toward assisted suicide and their experience confronting it. The description of the global outcome (4 items) and measurement of learning gains on the levels of knowledge, skills, and attitude were also surveyed (8 items). Data were collected by means of a 6-point Likert scale (completely wrong – completely right and completely disagree – completely agree) and descriptively analyzed. The comparative self-assessment gain (CSA gain) was computed using the formula: CSA gain=(µpre-µpost)/(µpre-1) x 100, making it possible to show CSA as a percentage for concrete learning objectives, based on a self-assessment in the post- and then-test.

The online course evaluation and the in-depth overall evaluation of student attitudes toward the two teaching methods based on semi-structured interviews are still underway and will be published later. The results presented here apply to the classroom-based course.

## Results

A total of 15 students (80% female) participated in the pilot project during the 2023 summer semester. The students were on average 22 years old (19-31 years) and ranged in semester level from the 4^th^ to the 10^th^.

A question about the legal and professional situation in Germany regarding assisted suicide and termination of life on request was answered correctly by a predominant majority of the students. Only the question about the provision of assisted suicide services was skipped over by 8 students, and one person incorrectly stated that termination of life on request can be offered as a service in Germany.

Regardless of any specific scenarios, the students were first asked about the medical role in the context of the wish to die. Attending to people desiring death and assisted suicide was viewed as a form of medical care by the majority of the students. All of the students found that termination of life on request was not medical care.

**Figure 2:**
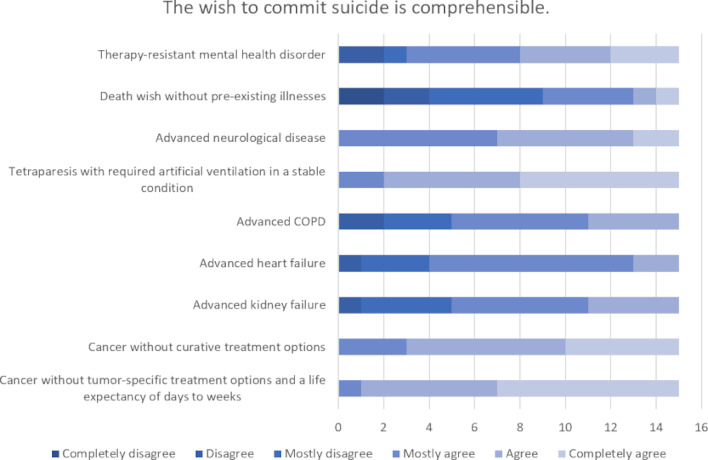
Evaluation of specific scenarios in terms of the comprehensibility of the desire for assisted suicide

**Figure 3:**
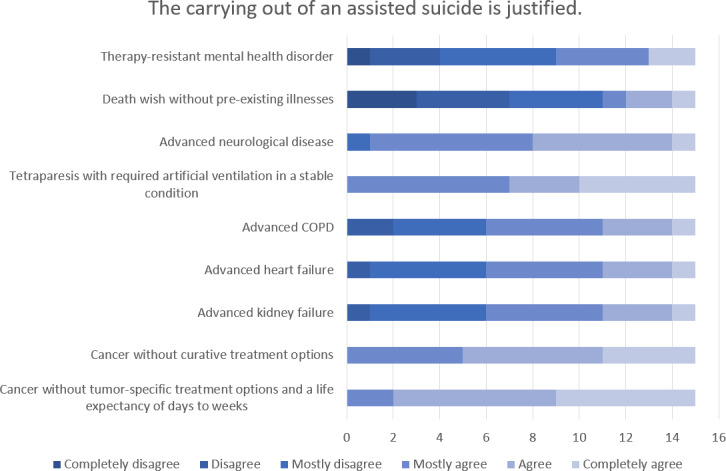
Evaluation of specific scenarios in terms of the comprehensibility of the desire for termination of life on request

**Figure 4:**
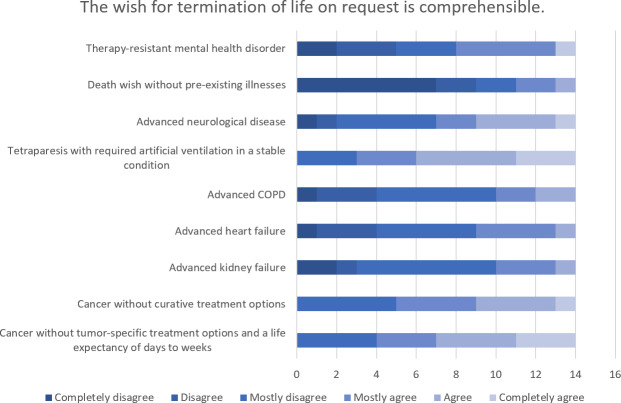
Evaluation of specific scenarios in terms of the justification for carrying out assisted suicide

**Figure 5:**
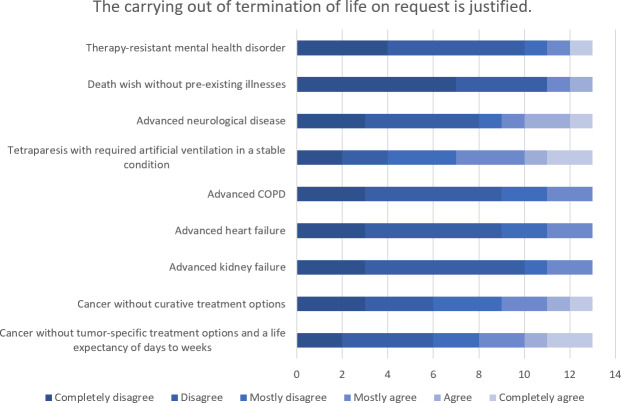
Evaluation of specific scenarios in terms of the justification for carrying out termination of life on request

The highest level of agreement concerning the comprehensibility of a wish to commit suicide (n=15) and the justification for carrying it out (n=15) was found for the scenarios of “cancer without tumor-specific treatment options and a life expectancy of day to weeks” followed by “tetraparesis with required artificial ventilation in a stable condition”. The highest level of rejection was seen for both comprehensibility and justification in the case of a person’s “desire to die in the absence of any relevant pre-existing conditions”. There was extreme ambivalence in the evaluation of “therapy-resistant mental health disorders”. The evaluation of the comprehensibility of a request for termination of life (n=14) and the justification for carrying it out (n=13) showed similar weighting in the individual scenarios, whereby the level of rejection was overall higher (see figure 2 (Fig. 2), figure 3 (Fig. 3), figure 4 (Fig. 4) and figure 5 (Fig. 5)).

When analyzing the Likert scales, the responses ranging from “completely right/completely agree – mostly right/ostly agree” were evaluated as agreement, while “mostly wrong/mostly disagree – completely wrong/completely disagree” were viewed as rejection or disagreement.

Prior to the seminar, the students stated that they did not feel well prepared as individuals or as newly licensed physicians for a confrontation with the desire to commit suicide (n=15: *disagreement* 100%).

The self-assessed level of preparedness in the role of “physician” increased as a result of the intervention (n=15: *agreement *67%) and in the role of an “individual person” (n=15: *agreement* 60%).

Students are frightened when they Imagine being asked by a patient (n=15: *agreement* 67%) or a loved one (n=15: *agreement* 100%) for help committing suicide.

After the intervention, this fear of receiving a request from a patient (n=15: *disagreement* 60%) is distinctly less strong than the fear of a receiving a request from a loved one (n=15: *disagreement* 7%).

The majority of the students were emotionally moved by the conversation with the simulated patient (n=15: *agreement *93%), but not burdened (n=15: *disagreement* 73%) or overwhelmed (n=15: *disagreement* 67%).

The image of a playing field (see figure 1 (Fig. 1)) was presented to the students for the purpose of taking a dynamic stance regarding their own role and demarcating personal “red boundaries” around it. Students rated this visual aid as helpful (n=15: *agreement* 80%).

The playing field serves to visualize personal boundaries in the context of the professional role. For example, each individual or medical team can define the uncrossable red lines in regard to specific scenarios, example cases, or specific issues; for instance, a boundary can be personal involvement or the absence of a terminal illness, etc.

To determine the specific course outcomes [6], the self-assessed gain in learning was calculated for 8 course objectives; a clear gain was reported for each of these objectives (see table 1 (Tab. 1)). The lowest gain was described for the learning objective “I take a desire for suicide expressed to me seriously”, in regard to which the students had already rated themselves as competent prior to the intervention. Students reported the largest gains in informing others about the palliative alternatives to suicide and in reflecting on their own emotions and boundaries.

## Discussion

The students describe a gain in learning within the context of the intervention that is approximately equal across the cognitive (W1-2), psychomotor (F1-3) and affective (H1-3) outcome dimensions. In particular, providing information about palliative alternatives in terms of suicide prevention and self-reflection as an aspect of self-care are rated as having large gains in learning.

The survey of the students concerning their attitudes toward the wish to commit suicide in general and in specific scenarios shows, in addition to a basically open and liberal view on this subject, a nuanced consideration of concrete medical situations (e.g., cancers without curative treatment options, desire to die without relevant pre-existing illness). A not yet published survey of physicians, some with many years of medical experience, shows the same tendency in the evaluation of the individual scenarios.

The evaluation of specific scenarios raises the question if a knowledge deficit exists here: Carrying out an assisted suicide, for example, in the case of tetraparesis with required artificial ventilation is perceived as justified by all of the respondents in the study. However, wouldn’t a discontinuation of the ventilation with measures to relieve symptoms be a case of “allowing the dying process to happen” the most obvious way to end the patient's life, as wished for? Suicide would not be necessary.

Other scenarios, in which there is a high degree of agreement about the wish for assisted suicide and carrying it out, describe situations that are often accompanied by a need for palliative care. There is a large overlap between the comprehensibility of suicidal wishes and palliative care patients, which emphasizes the responsibility of palliative medicine to participate in the discussions about assisted suicide.

The students report inadequate preparation for the confrontation with requests for assisted suicide and thus have the associated fears and concerns. The double role deliberately selected for the students results, on the one hand, in a higher emotional involvement, but is also meant to enable them to have a more authentic empathy in the situation, since the role of family member is more familiar to them at this point in their education than the role of the attending physician. As a result of a short, interactive and practice-based educational intervention it is possible to address and reduce these fears in relation to the future medical role, whereby this effect is larger in the role of the attending physician.

Responding to requests for assisted suicide in the role of medical doctor is unique in so far that the legal framework provides great freedom to act but also conveys a great measure of responsibility. Contrary to “conventional” medical treatment, no orientation around the normal rules of diagnosis is possible here, the most decisive element is only the patient’s wish.

## Conclusion

Despite many limitations (small cohort, voluntary participation, short intervention, focus only on palliative medicine), this project report suggests that there is a need in medical education regarding the response to suicide requests. This paper describes the fear of assuming responsibility and backs up the question about whether or not undergraduate medical education in its current form is sufficiently prepared to meet this responsibility [7]. The international literature shows the influence of intrinsic and extrinsic factors (e.g., educational content and setting) on professional identity formation [8], such that being empowered to take on a responsibility can help physicians to better prepare themselves for difficult decisions and challenging medical situations.

## Authors’ ORCIDs

- Alexandra Scherg: [0000-0002-8778-7429]
- Martin Lemos: [0000-0002-0788-2400]

## Funding

This project took place as part of the Erasmus+ ELPIS project and was paid for out of the corresponding funds (KA220-HED-AF6D681C).

## Competing interests

The authors declare that they have no competing interests. 

## Figures and Tables

**Table 1:**
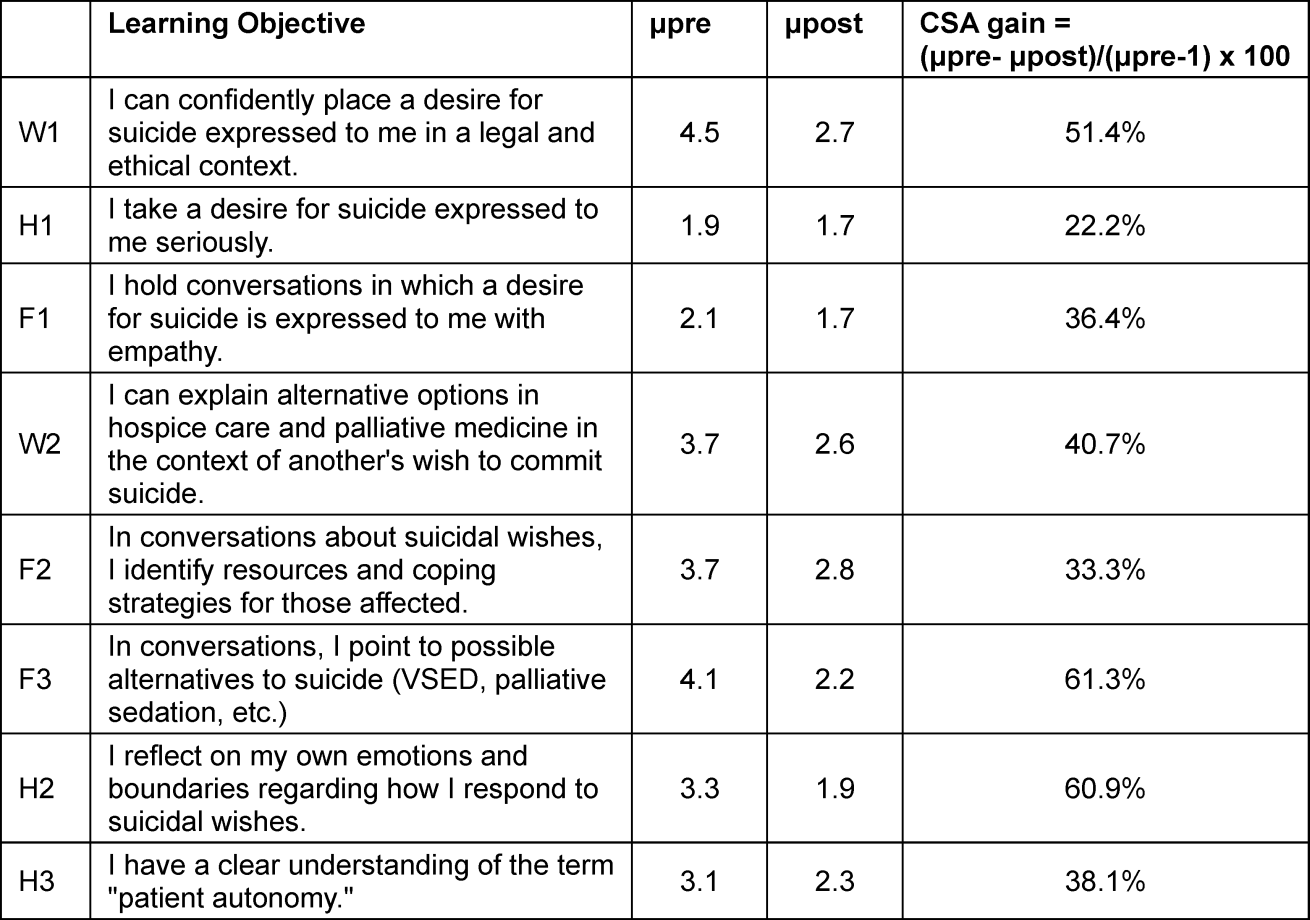
Gain in learning for specific course objectives in the dimensions of knowledge (W1-2), skills (F1-3) und attitude (H1-3)
